# Genotypic and Phenotypic Detection of AmpC β-lactamases in *Enterobacter* spp. Isolated from a Teaching Hospital in Malaysia

**DOI:** 10.1371/journal.pone.0150643

**Published:** 2016-03-10

**Authors:** Fatin Izzati Mohd Khari, Rina Karunakaran, Roshalina Rosli, Sun Tee Tay

**Affiliations:** Department of Medical Microbiology, Faculty of Medicine, University of Malaya, 50603, Kuala Lumpur, Malaysia; Second University of Naples, ITALY

## Abstract

**Objectives:**

The objective of this study was to determine the occurrence of chromosomal and plasmid-mediated β-lactamases (AmpC) genes in a collection of Malaysian isolates of *Enterobacter* species. Several phenotypic tests for detection of AmpC production of *Enterobacter* spp. were evaluated and the agreements between tests were determined.

**Methods:**

Antimicrobial susceptibility profiles for 117 *Enterobacter* clinical isolates obtained from the Medical Microbiology Diagnostic Laboratory, University Malaya Medical Centre, Malaysia, from November 2012—February 2014 were determined in accordance to CLSI guidelines. AmpC genes were detected using a multiplex PCR assay targeting the MIR/ACT gene (closely related to chromosomal EBC family gene) and other plasmid-mediated genes, including DHA, MOX, CMY, ACC, and FOX. The AmpC β-lactamase production of the isolates was assessed using cefoxitin disk screening test, D69C AmpC detection set, cefoxitin-cloxacillin double disk synergy test (CC-DDS) and AmpC induction test.

**Results:**

Among the *Enterobacter* isolates in this study, 39.3% were resistant to cefotaxime and ceftriaxone and 23.9% were resistant to ceftazidime. Ten (8.5%) of the isolates were resistant to cefepime, and one isolate was resistant to meropenem. Chromosomal EBC family gene was amplified from 36 (47.4%) *E*. *cloacae* and three (25%) *E*. *asburiae*. A novel *bla*_DHA_ type plasmid-mediated AmpC gene was identified for the first time from an *E*. *cloacae* isolate. AmpC β-lactamase production was detected in 99 (89.2%) of 111 potential AmpC β-lactamase producers (positive in cefoxitin disk screening) using D69C AmpC detection set. The detection rates were lower with CC-DDS (80.2%) and AmpC induction tests (50.5%). There was low agreement between the D69C AmpC detection set and the other two phenotypic tests. Of the 40 isolates with AmpC genes detected in this study, 87.5%, 77.5% and 50.0% of these isolates were positive by the D69C AmpC detection set, CC-DDS and AmpC induction tests, respectively.

**Conclusions:**

Besides MIR/ACT gene, a novel plasmid-mediated AmpC gene belonging to the DHA-type was identified in this study. Low agreement was noted between the D69C AmpC detection set and two other phenotypic tests for detection of AmpC production in *Enterobacter* spp. As plasmid-mediated genes may serve as the reservoir for the emergence of antibiotic resistance in a clinical setting, surveillance and infection control measures are necessary to limit the spread of these genes in the hospital.

## Introduction

*Enterobacter* spp. has been recognised as a major nosocomial pathogen which causes outbreaks and bloodstream infections [1]. Amongst the species in the genus *Enterobacter*, *Enterobacter cloacae* and *Enterobacter aerogenes* are two most common species isolated from clinical specimens [2]. High occurrence (more than 30%) of cephalosporin resistance has been noted for *Enterobacter* isolates from several parts of the world including USA, Europe, Middle East and Asia [3–8]. Resistance of *Enterobacter* spp. to broad-spectrum cephalosporins such as cefotaxime, ceftazidime, and ceftriaxone has been attributed to the production of AmpC β-lactamase (AmpC), which confers resistance to aminopenicillins, cephalosporins, oxyimino-cephalosporins (ceftriaxone, cefotaxime and ceftazidime), cephamycins (cefoxitin and cefotetan) and monobactam (aztreonam) [9]; isolates sensitive to third-generation cephalosporins can also become resistant while on treatment [9].

In the *Enterobacteriaceae*, AmpC enzymes are encoded by either chromosomal or plasmid-mediated genes. Majority of the chromosomal AmpC β-lactamases can be found in *Enterobacter*, *Serratia*, *Pseudomonas*, *Acinetobacte*r and *Citrobacter* spp. [10]. Plasmid-mediated AmpC β-lactamases, including MIR/ACT (associated with EBC family gene), DHA, MOX, CIT, ACC, and FOX [11] are derived from chromosomal AmpC genes of *Enterobacteriaceae* which display structural and functional similarity to their chromosomal origins [12]. MIR-1 [13] and ACT-1 [14] are plasmid-encoded cephalosporinases derived from *E*. *cloacae*. The first isolation of a *bla*_DHA_ (DHA7) gene from an *E*. *cloacae* isolate has been recently reported in Spain [15]. The overproduction of AmpC β-lactamases in *Enterobacter* spp. confers resistance to most β-lactam antibiotics, except for carbapenems and fourth-generation cephalosporins (cefepime), although caution is suggested in the use of the latter for therapy [9].

The exact prevalence of AmpC, particularly those mediated by plasmids in *Enterobacteriaceae* is still largely unknown due to the lack of simple and valid detection methods [16]. Currently, there are no recommendations for routine AmpC β-lactamase testing for *Enterobacter* spp. [17]. Nevertheless, surveillance and monitoring activity is still important in this era of increasing antibiotic resistance as failure in the detection of antibiotic resistant determinants may result in the dissemination of resistant bacteria and indirectly, complicates the management and treatment of patients.

Cefoxitin resistance has been used for screening of AmpC β-lactamases producers in the *Enterobacteriaceae*. Although cefoxitin is stable against the activity of multiple β-lactamases such as TEM-1, -2, and SHV-1 [18], its resistance may also be mediated by alterations to outer membrane permeability [19, 20]. Disk-based assays, using a variety of combinations of antibiotic substrates (such as cloxacillin) and inhibitors (boronic acid compounds) have been developed [21–24]. A commercial test kit, D69C AmpC detection set (MASTDISCS™ID, UK) has been validated for detection of both chromosomal and plasmid-mediated AmpC β-lactamase in the *Enterobacteriaceae* [25]. The cefoxitin-cloxacillin double disk synergy (CC-DDS) [26] and AmpC induction tests [27] have been developed for detection of AmpC-producing β-lactamase isolates in *Enterobacteriaceae* naturally lacking chromosomal AmpC beta-lactamases such as *Klebsiella* spp., *Salmonella* spp., and *Proteus mirabilis*. However, there have not been any intensive studies on their use for *Enterobacter* spp.

A multiplex PCR assay has been described for specific detection of six families of plasmid-acquired AmpC genes [11]. The PCR assay has also been used for detection of chromosomal AmpC gene from *Enterobacter* isolates due to the high sequence similarity with MIR/ACT gene [28, 29]. Little information is available on the occurrence of chromosomal and plasmid-mediated AmpC genes for Malaysian clinical isolates of *Enterobacter* spp. Hence, this study was designed to determine the occurrence of these genes in several species of *Enterobacter* isolated from Malaysian patients and to evaluate AmpC β-lactamase production of the isolates using currently available phenotypic assays, i.e., D69C AmpC detection set, cefoxitin-cloxacillin double disk synergy test (CC-DDS) and AmpC induction test.

## Materials and Methods

### Ethical statement

This study has obtained approval from the Medical Ethics Committee, University Malaya Medical Center, Kuala Lumpur, Malaysia (MECID. No:20144–87). The need to have written or verbal informed consent from patients was not required because of the retrospective nature of the study. Patient records/information was anonymized and de-identified prior to analysis.

### Bacterial isolates

A total of 117 non-duplicate and non-sequential *Enterobacter* isolates were obtained from the Medical Microbiology Diagnostic Laboratory, University Malaya Medical Centre, Malaysia. Collection of *Enterobacter* isolates from blood cultures began on November 2012, while isolates from other specimens were obtained from July 2013 to February 2014. The isolates originated from various clinical specimens, i.e blood (n = 63), respiratory specimens (n = 19), swabs from various sites (n = 13), tissues (n = 8), urine (n = 7) and others (n = 7). The full list and origin of *Enterobacter* isolates investigated in this study are shown in S1 Table. The isolates were identified to the species level using Matrix Assisted Laser Desorption Ionization–Time of Flight mass spectrometry (MALDI-TOF MS) (Bruker Daltonics, Bremen, Germany), as described previously by Clark et al. [30]. Of the 117 isolates of *Enterobacter* spp., 76 (64.9%) isolates were *E*. *cloacae*, 27 (23.1%) were *E*. *aerogenes*, 12 (10.3%) were *E*. *asburiae* and two (1.7%) were *E*. *gergoviae* (currently known as *Pluralibacter gergoviae)*.

### Antimicrobial susceptibility testing

The antibiotic susceptibilities of the isolates were determined using Kirby Bauer’s disk diffusion method as described in the guidelines of Clinical and Laboratory Standard Institute (CLSI) [31]. The antibiotic disks used were cefuroxime (30 μg), ceftriaxone (30 μg), ceftazidime (30 μg), cefotaxime (30 μg), cefepime (30 μg), trimethoprim-sulfamethoxazole (25 μg), gentamicin (30 μg), ciprofloxacin (30 μg), amikacin (30 μg), meropenem (30 μg) and piperacillin-tazobactam (100/10 μg). Results were interpreted as sensitive, intermediate and resistant according to CLSI guidelines [31]. For cefepime, an interpretation for Susceptible Dose Dependent (SDD) was also included. *Escherichia coli* ATCC 25922 strain was used as the quality control strain for the antibiotic susceptibility tests.

### Molecular analyses of AmpC genes

The presence of plasmid-mediated AmpC genes was investigated using a multiplex PCR assay targeting MIR/ACT (closely related with EBC family gene), DHA, MOX, CIT, ACC, and FOX, as described by Pérez-Pérez and Hanson [11]. If an AmpC gene is detected by PCR that is specific for the species concerned, it is considered as chromosomal [9, 16]. Hence, any MIR/ACT gene detected from the isolates in this study was regarded as chromosomal AmpC gene. Each isolate was cultured on nutrient agar and incubated at 37°C for 18–24 hr. Colonies were suspended in 500 μl sterile distilled water, heated at 95°C for 10 min and cooled in ice for 10 min. The lysate was briefly vortexed, and centrifuged at 13,000 rpm for 10 min. The supernatant was used as the DNA template [11].

A 25 μl PCR mixture containing 0.2 μl of DNA template, 2.5 μl of 10X *Taq* buffer, 0.5 μl of 10 mM dNTPs, 0.3 μM primers MOXMF, MOXMR, CITMF, CITMR, DHAMF and DHAMR; 0.25 μM primers ACCMF, ACCMR, EBCMF and EBCMR; 0.2 μM primers FOXMF and FOXMR and 1 U of *Taq* DNA polymerase (Fermentas, Thermo Scientific, USA), was prepared. PCR was performed in a Veriti 96-well thermal cycler (Applied Biosystems, USA) at 94°C for 3 min followed by 25 cycles of 94°C for 30 sec, 64°C for 30 sec, and 72°C for 1 min with a final extension at 72°C for 7 min. Amplified products were then analyzed by electrophoresis on a 2% agarose gel at 100V for 45 min, stained with GelRed™ (Biotium Inc., USA), and the image was captured using InGenius gel documentation system (Syngene, England). A 100bp DNA ladder (Promega, USA) was used as a size marker. The boil lysate of DHA-1 carrying *K*. *pneumoniae* strain M40 was used as the positive control [32], while distilled water was used as the negative control. To determine the full length sequence of DHA positive isolate, primers obtained from Pérez-Llarena et al. [15] with slight modification, DHAfw (5’-ATGAAAAAATCGTTATCTGCAACAC-3’) and DHArv (5’-TTATTCCAGTGCACTCAAAATAGC-3’) were used. The amplification include initial denaturation at 95°C for 15 min, followed by 30 cycles of denaturation at 94°C for 30 sec, annealing at 54°C for 90 sec and extension at 72°C for 1 min, and a final extension step at 72°C for 10 min.

Purified PCR products were subjected to sequence determination using respective primers. Sequencing was performed on an ABI PRISM 377 Genetic Analyzer (Applied Biosystem, USA). BLAST analysis was performed to search for homologous sequences in the GenBank database.

### Phenotypic detection of AmpC β-lactamases

Cefoxitin disks (30 μg) were used for screening of AmpC-producing isolates, as described by Polsfuss et al. [26]. Isolates exhibiting inhibition zones with diameter ≤ 18 mm were considered positive for AmpC screening and were subjected to further testing using D69C AmpC detection set (MASTDISCS™ID, UK), cefoxitin-cloxacillin double disk synergy test (CC-DDS) [26], and AmpC induction tests [27]. A DHA-1 carrying *K*. *pneumoniae* strain (M40) was used as the positive control strain for all the tests [32].

1. D69C AmpC detection set. This test uses cefpodoxime as a screening agent for detection of both plasmid-mediated and chromosomal AmpC, with the presence of AmpC inducer or inhibitor (exact formulation undisclosed). The interference from any ESBL on the test is minimized using an ESBL inhibitor. The detection set comprises three disks: disk A (cefpodoxime 10 μg and AmpC inducer), disk B (cefpodoxime 10 μg, AmpC inducer and ESBL inhibitor) and disk C (cefpodoxime 10 μg, AmpC inducer, ESBL inhibitor and AmpC inhibitor). The test was performed and interpreted in accordance to the manufacturer’s instructions (MASTDISCS™ID, UK). An isolate is interpreted as ‘AmpC positive’ if the zone of inhibition of the disk C exceeded both disks A and B by at least 5 mm, and ‘AmpC negative’ if all zone sizes differed by ≤ 3 mm. If the zone of inhibition of both disk B and C exceeded that of disk A by at least 5 mm, and the zone sizes of disk B and C had a difference of ≤ 4 mm, the isolate is regarded as ‘AmpC negative but exhibits a different resistance mechanism’ [25].
2. Cefoxitin-cloxacillin double disk synergy test (CC-DDS). This test was conducted based on the inhibitory effect of cloxacillin on AmpC production. The isolates were inoculated on Mueller Hinton agar (Oxoid, UK) as described previously [26]. Cefoxitin/cloxacillin disks (230 μg) and cefoxitin disk (30 μg) were used in this study. A difference of ≥ 4 mm in the inhibition zones of cefoxitin/cloxacillin and cefoxitin disks was an indication of AmpC production [26].
3. AmpC induction test. Clavulanate is used as an inducer for detection of plasmid-mediated AmpC β-lactamase for *Enterobacteriaceae* in a double-disk test method [27]. Disk containing ceftazidime (30 μg) and aztreonam (30 μg) were placed 15 mm edge to edge from an amoxicillin-clavulanate disk (30 μg) on Mueller-Hinton agar plates. The test was interpreted as positive when flattening zones of inhibition of ceftazidime and aztreonam disk adjacent to the amoxicillin-clavulanate disk were observed.

The percentage of agreement for CC-DDS and AmpC induction tests against D69C AmpC detection set were determined. The tests were considered as in agreement with D69C AmpC detection set if the CC-DDS or AmpC induction test showed similar results as D69C AmpC detection set for detection of AmpC β-lactamase.

## Results

### Antimicrobial susceptibility testing

Overall, of 117 *Enterobacter* spp. investigated in this study, 39.3% of the isolates were resistant to both cefotaxime and ceftriaxone while 23.9% of the isolates were resistant to ceftazidime (Table 1). Another 7.7%, 2.6% and 11.1% of the isolates demonstrated intermediate resistance to cefotaxime, ceftriaxone and ceftazidime, respectively. For cefuroxime, although 59.8% of the isolates of *Enterobacter* species were sensitive, the result was unreliable due to the intrinsic resistance of *Enterobacter* spp. to cefuroxime [31]. Ten (8.5%) *Enterobacter* isolates were resistant to cefepime, another 13.7% were ‘susceptible dose dependent’.

**Table 1:** Antimicrobial susceptibility profiles of *Enterobacter* spp. investigated in this study.

Antibiotics	No. (%) *Enterobacter* isolates												No. (%) resistant isolates
	*E*. *cloacae* (n = 76)			*E*. *aerogenes* (n = 27)			*E*. *asburiae* (n = 12)			*E*. *gergoviae* (n = 2)			
	S	I	R	S	I	R	S	I	R	S	I	R	
**CXM 30**	39 (51.3)	-	37 (48.7)	19 (70.4)	1 (3.7)	7 (25.9)	10 (83.3)	2 (16.7)	-	2 (100.0)	-	-	44 (37.6)
**CRO 30**	39 (51.3)	-	37 (48.7)	18 (66.7)	2 (7.4)	7 (25.9)	10 (83.3)	1 (8.3)	1 (8.3)	1 (50.0)	-	1 (50.0)	46 (39.3)
**CAZ 30**	43 (56.6)	10 (13.2)	23 (30.3)	20 (74.1)	3 (11.1)	4 (14.8)	12 (100.0)	-	-	1 (50.0)	-	1 (50.0)	28 (23.9)
**CTX 30**	32 (42.1)	6 (7.9)	38 (50.0)	17 (63.0)	3 (11.1)	7 (25.9)	12 (100.0)	-	-	1 (50.0)	-	1 (50.0)	46 (39.3)
**FEP 30**	53 (69.7)	*15 (19.7)	8 (10.5)	24 (88.9)	*1(3.7)	2 (7.4)	12 (100.0)	-	-	2 (100.0)	-	-	10 (8.5)
**SXT 25**	45 (59.2)	2 (2.6)	29 (38.2)	25 (92.6)	1 (3.7)	1 (3.7)	11 (91.7)	-	1 (8.3)	1 (50.0)	-	1 (50.0)	32 (27.4)
**CN 10**	59 (77.6)	1 (1.3)	16 (21.1)	26 (96.3)	-	1 (3.7)	12 (100.0)	-	-	2 (100.0)	-	-	17 (14.5)
**CIP 5**	71 (93.4)	-	5 (6.6)	25 (92.6)	2 (7.4)	-	12 (100.0)	-	-	2 (100.0)	-	-	5 (4.3)
**AK 30**	69 (90.8)	3 (3.9)	4 (5.3)	26 (96.3)	-	1 (3.7)	12 (100.0)	-	-	2 (100.0)	-	-	5 (4.3)
**MRP 10**	76 (100.0)	-	-	24 (88.9)	2 (7.4)	1 (3.7)	12 (100.0)	-	-	2 (100.0)	-	-	1 (0.9)
**TZP 110**	59 (77.6)	6 (7.9)	11 (14.5)	23 (85.2)	3 (11.1)	1 (3.7)	12 (100.0)	-	-	2 (100.0)	-	-	12 (10.3)
**FOX 30**	4 (5.3)	1 (1.3)	71 (93.4)	1 (3.7)	-	26 (96.3)	-	-	12 (100.0)	1 (50.0)	-	1 (50.0)	110 (94.0)

Abbreviation

CXM 30, Cefuroxime (30 μg)

CRO 30, Ceftriaxone (30 μg)

CAZ 30, Ceftazidime (30 μg)

CTX 30, Cefotaxime (30 μg)

FEP 30, Cefepime (30 μg)

SXT 25, trimethoprim-sulfamethoxazole (25 μg)

CN 10, Gentamicin (10 μg)

CIP 5, Ciprofloxacin (5 μg)

AK 30, Amikacin (30 μg)

MRP 10, Meropenem (10 μg)

TZP 110, Piperacillin+Tazobactam (110 μg)

FOX 30, Cefoxitin (30μg).

Abbreviation

S, Sensitive

I, Intermediate

SDD (for cefepime*), Susceptible Dose Dependent

R, Resistant.

Apart from the two isolates of *E*. *gergoviae* of which 50% were resistant to cefotaxime, ceftriaxone and ceftazidime, *E*. *cloacae* was the species demonstrating the next highest resistance to the third-generation cephalosporins, with 38 (50%), 37 (48.7%) and 23 (30.3%) of the 76 isolates being resistant to cefotaxime, ceftriaxone and ceftazidime, respectively. Among the 12 isolates of *E*. *asburiae*, only one isolate (8.3%) was resistant to ceftriaxone and the remaining isolates were sensitive to ceftazidime, cefotaxime and cefepime.

Of the 117 *Enterobacter* isolates, 110 (94%) were sensitive to ciprofloxacin, 82 (70.1%) were sensitive to trimethoprim-sulfamethoxazole, 96 (82.1%) were sensitive to piperacillin-tazobactam, while 109 (93.2%) and 99 (84.6%) were sensitive to amikacin and gentamicin, respectively. High occurrence of resistant isolates against ceftriaxone (48.7 vs 25.9%), cefotaxime (50.0 vs 25.9%), ceftazidime (30.3 vs 14.8%), trimethoprim-sulfamethoxazole (38.2 vs 3.7%) and gentamicin (21.1 vs 3.7%) was noted in the *E*. *cloacae*, as compared to *E*. *aerogenes* isolates. Majority of *Enterobacter* isolates (97.4%) were sensitive to meropenem; one isolate of *E*. *aerogenes* was resistant and two were intermediate (Table 1). The antibiotic susceptibility profiles of the isolates investigated in this study is shown in S1 Table.

### PCR detection of AmpC genes

A total of 40 (34.2%) of 117 *Enterobacter* isolates were positive for AmpC gene (Table 2), of which 39 (33.3%) isolates were detected with MIR/ACT gene. *E*. *cloacae* demonstrated the highest detection rate of MIR/ACT gene in 36 (47.4%) of 76 isolates, followed by *E*. *asburiae*, with three (25.0%) of the 12 isolates detected for the gene. MIR/ACT gene was not detected in *E*. *aerogenes* and *E*. *gergoviae*.

**Table 2:** The occurrence of AmpC genes in *Enterobacter* isolates.

*Enterobacter* species	No. (%) isolates carrying		Total (%)
	DHA	*MIR/ACT	
***E*. *cloacae* (n = 76)**	1 (1.3)	36 (47.4)	37 (48.7)
***E*. *aerogenes* (n = 27)**	0 (0.0)	0 (0.0)	0 (0.0)
***E*. *asburiae* (n = 12)**	0 (0.0)	3 (25.0)	3 (25.0)
***E*. *gergoviae* (n = 2)**	0 (0.0)	0 (0.0)	0 (0.0)
**Total (n = 117)**	**1 (0.9)**	**39 (33.3)**	**40 (34.2)**

*MIR/ACT gene is known to be originated from *E*. *cloacae* chromosome.

Sequence analysis of the amplified fragments from randomly selected isolates from each *Enterobacter* species confirmed the identification of MIR/ACT genes. The amplified fragment obtained from a representative *E*. *cloacae* isolate shows the highest sequence similarity (99%, 212/213 nucleotides) with the AmpC gene of *E*. *cloacae* strain P99 (Genbank accession no. X07274, [28]), while analysis of a partial fragment amplified from an *E*. *asburiae* isolate demonstrated the highest sequence similarity (97%, 196/203 nucleotides) matching with the AmpC gene (AJ311172) of the *E*. *asburiae* type strain.

A plasmid-mediated AmpC gene (DHA-type) was detected from an *E*. *cloacae* isolate obtained from the urine culture of a patient. The almost full length sequence (1073 bp) of the DHA gene was identical to DHA type 1 (Genbank accession no. Y16410), except for a mutation (G733T) which resulted with an amino acid change (A245S). Compared to DHA-type 7 gene which was derived from an *E*. *cloacae* strain from Spain (Genbank accession no. HQ456945), the sequence has an additional nucleic acid (C1019T) and amino acid (F340S) change.

### Phenotypic detection of AmpC β-lactamases

Table 3 shows the detection of AmpC production using three phenotypic tests. Cefoxitin disk screening test for 117 *Enterobacter* isolates revealed 111 (94.9%) isolates as potential AmpC β-lactamase producers. Positive results using D69C AmpC detection set were obtained from 89.2% (99/111) isolates. Using the CC-DDS test, 89 (80.2%) of the 111 isolates were positive, while 56 (50.5%) isolates were positive by the AmpC induction test.

**Table 3:** Detection of AmpC production in *Enterobacter* spp. by phenotypic tests and the percentages of agreement between tests (in 111 isolates positive by the cefoxitin screening test).

*Enterobacter* species	No. (%) positive isolates			No. (%) isolates in agreement	
	D69C AmpC detection set	CC-DDS	AmpC induction test	between D69C AmpC detection set and CC-DDS test	between D69C AmpC detection set and AmpC induction test
*E*. *cloacae* (n = 72)	66 (91.7)	54 (75.0)	38 (52.8)	50 (69.4)	38 (52.8)
*E*. *aerogenes* (n = 26)	22 (84.6)	24 (92.3)	12 (46.2)	22 (84.6)	12 (46.2)
*E*. *asburiae* (n = 12)	11 (91.7)	11 (91.7)	6 (50.0)	10 (83.3)	7 (58.3)
*E*. *gergoviae* * (n = 1)	0 (0.0)	0 (0.0)	0 (0.0)	1 (100.0*)	1 (100.0*)
Total (n = 111)	99 (89.2)	89 (80.2)	56 (50.5)	83 (74.8)	58 (52.3)

Abbreviation

FOX, cefoxitin (30μg)

CC-DDS, cefoxitin-cloxacillin double disk.

* The percentage agreement between assays for *E*. *gergoviae* was not reliable as only one isolate was available for comparison.

The percentage of agreement between CC-DDS test against D69C AmpC detection set and AmpC induction test against D69C AmpC detection set was determined, as shown in Table 3. CC-DDS test demonstrated higher percentage of agreement (74.8%) with D69C AmpC detection set, as compared to 52.3% agreement noted with the AmpC induction test.

### Comparison of phenotypic test results with PCR detection of AmpC β-lactamase genes

A total of 71 (64.0%) of the 111 isolates positive in the cefoxitin disk screening test did not have any AmpC genes detected in this study, yet exhibited positive reactions in the D69C AmpC detection test (n = 64), CC-DDS (n = 58) and AmpC induction test (n = 36). Of the six isolates negative in the cefoxitin disk screening test, none were positive for AmpC genes by the multiplex PCR assay.

Table 4 shows the comparison of the results obtained from PCR assays and three phenotypic tests. Of the 39 isolates with MIR/ACT genes detected, D69C AmpC detection set detected 34 (87.2%) isolates, while CC-DDS test detected 30 (76.9%) isolates. AmpC induction test demonstrated the lowest detection rate (48.7%). The only isolate carrying DHA gene was detected by all three phenotypic tests. Of the five isolates with MIR/ACT genes detected but negative in the D69C AmpC detection set, all were positive by the CC-DDS test while only one was positive by the AmpC induction test. The results of phenotypic and genotypic detection of AmpC β-lactamases are shown in S1 Table.

**Table 4:** Comparison of phenotypic tests results with PCR detection of AmpC genes.

AmpC gene	No. (%) detected by phenotypic tests		
	D69C AmpC detection set	CC-DDS test	AmpC induction test
MIR/ACT (n = 39)	34 (87.2)	30 (76.9)	19 (48.7)
DHA (n = 1)	1 (100.0)	1 (100.0)	1 (100.0)
Total AmpC detected (n = 40)	35 (87.5)	31 (77.5)	20 (50.0)

Abbreviation

CC-DDS, cefoxitin-cloxacillin double disk test.

## Discussion

Approximately 40% *Enterobacter* isolates investigated in this study were resistant to cefotaxime and ceftriaxone while 23.9% were resistant to ceftazidime (Table 1). Although some of the isolates were sensitive to the third-generation cephalosporins, it is known that *Enterobacter* spp., may develop resistance to these agents during treatment and should be avoided in the treatment of serious *Enterobacter* infections [33]. Only 8.5% of the isolates were resistant to cefepime, the fourth-generation cephalosporin, however another 13.7% were ‘susceptible dose dependent’ following the new CLSI interpretation criteria for cefepime [31].

Although 59.8% of the *Enterobacter* isolates in this study demonstrated susceptibility to cefuroxime by disk-diffusion method, the results should be interpreted with caution due to the fact that *E*. *cloacae* and *E*. *aerogenes* are intrinsically resistant to cefuroxime [31]. CLSI [31] states that a small percentage of *Enterobacter* isolates may appear sensitive to cefuroxime due to ‘method variation, mutation, or low levels of resistance expression’. Similarly, although majority of the *Enterobacter* spp. in this study were found to be sensitive to piperacillin-tazobactam (82.1%), it is usually avoided for treatment as it has the potential for AmpC induction [9] and poor treatment outcomes have been previously shown in animals infected with *E*. *coli* producing plasmid-mediated AmpC β-lactamase [34]. Besides the high resistance of *E*. *cloacae* isolates against ceftriaxone and cefotaxime, the resistance against trimethoprim-sulfamethoxazole, piperacillin-tazobactam and gentamicin was particularly higher in *E*. *cloacae* isolates compared to other *Enterobacter* spp. (excluding two *E*. *gergoviae* isolates, of which one was resistant to the third-generation cephalosporins and trimethoprim-sulfamethoxazole).

The detection of MIR/ACT AmpC gene in 36 (47.4%) of 76 *E*. *cloacae* isolates (Table 2) in this study is not surprising as the gene has been reported as the most dominant AmpC gene in 35 out of the 45 *E*. *cloacae* isolates causing intra-abdominal infections from the Asia-Pacific area [29]. The high occurrence of this gene in *Enterobacteriaceae* (in 75% of isolates tested) has also been reported in Thailand [35]. The presence of EBC family gene in *E*. *aerogenes* has been documented [36], however; it was not detected in this study. So far, the EBC family gene has not been reported for *E*. *gergoviae*. The negative findings from PCR detection of MIR/ACT gene in some *E*. *cloacae* and all the *E*. *aerogenes* isolates in the present study may be caused by the lack of AmpC gene or sequence variability of the AmpC genes which resulted in the gene not amplifiable by the PCR primers used in this study.

Plasmid-mediated AmpC β-lactamases pose a big challenge to infection control due to the fact that the AmpC gene can be expressed in larger amounts and has high transmissibility to other bacterial species [37]. Plasmid-mediated AmpC genes that have been reported in *Enterobacter* species include DHA-type which has been reported in Spain [15], Taiwan [38] and Algeria [39]; CIT and ACC genes in Thailand [35], and FOX/MOX genes in Egypt [40]. This report represents the first identification of a novel DHA gene in a Malaysian *E*. *cloacae* isolate. The finding of a DHA-type plasmid-mediated gene in *E*. *cloacae* in this study, as well as in a variety of *Enterobacteriaceae* isolates including *E*. *coli* and *K*. *pneumoniae* in the Southeast Asia suggests a rapid spread and evolution of the *bla*_DHA_ type plasmid-mediated AmpC gene in this region [29, 32].

Majority (94.9%) of the *Enterobacter* isolates in this study were potential AmpC producers, based on the finding from cefoxitin disk screening test. As cefoxitin resistance can be affected by the reduced outer membrane permeability of the isolates [41], additional genotypic and phenotypic tests are necessary for confirmation of AmpC production. One of the reasons for the exact prevalence of AmpC production in *Enterobacter* spp. remains unknown is probably due to the lack of reliable laboratory methods for testing. Phenotypic detection of plasmid-mediated AmpC β-lactamases has been described to have poor specificity and is not advisable for routine detection of these β-lactamases [16]. The D69C AmpC detection set (MASTDISCS™ID, UK) used in this study has been validated using a large number of *Enterobacteriaceae* [25]. Among the phenotypic tests used for AmpC detection, the D69C AmpC detection set detected the highest number (89.2%) of *Enterobacter* isolates as compared to the CC-DDS (80.2%) and AmpC induction tests (50.5%). CC-DDS test demonstrated higher agreement (74.8%) with the D69C AmpC detection set, while the AmpC induction test showed a low agreement (52.3%) with D69C AmpC detection set. Additionally, the results of AmpC induction test were subjective and difficult to interpret. The flattening of the inhibition zone of ceftazidime/aztreonam disks towards the amoxicillin-clavulanate disk can be interfered by the presence of ESBL enzymes [27]. Moreover, clavulanate is not the most potent AmpC inducer compound [9]. However, this test has been used successfully for detection of DHA-1 plasmid-mediated gene in *K*. *pneumoniae* [27, 32], as well as the DHA gene identified in this study.

A high number (n = 71) of isolates positive in the cefoxitin disk screening test in this study did not have any AmpC genes detected by the multiplex PCR assay, yet some of these isolates exhibited positive reactions either in the D69C detection set, CC-DDS or AmpC induction tests. Further genotypic experiments are required to determine the mechanisms involved in the phenotypic expression of AmpC β-lactamase production in these isolates. Additionally, detection of AmpC genes from *Enterobacter* isolates could be further improved by the use of species-specific primers in the PCR assays since genetic diversity of chromosomal AmpC β-lactamases from *Enterobacter* isolates have been reported [28, 42].

Establishing the prevalence of AmpC β-lactamase production in the *Enterobactericeae* is critical as part of the surveillance and monitoring activity of antibiotic resistance for infection control. The genotypic approach used in this study has facilitated the detection of a plasmid-mediated AmpC gene (a novel DHA-type) besides the chromosomal AmpC gene (MIR/ACT) in *Enterobacter* spp. The phenotypic detection of AmpC β-lactamase production has been hampered by the lack of validated methods, as suggested by the low agreement between the D69C AmpC detection set and the other phenotypic tests. The emergence of plasmid-mediated AmpC and ESBL β-lactamase producing *Enterobacter* spp. may pose potential risk to the spread of antibiotic resistance in the clinical settings. As plasmid-mediated genes may serve as the reservoir for the emergence of antibiotic resistance in a clinical setting, surveillance and infection control measures are essential to limit the spread of these organisms in the hospital.

## Supporting Information

S1 Table The full list of *Enterobacter* isolates investigated in this study and the results of antibiotic susceptibility, phenotypic and genotypic detection of AmpC β-lactamases. (XLSX)
